# Supplementary material for: Resolving the full spectrum of human genome variation using Linked-Reads
Source: Genome Res. 2019 Apr;29(4):635–45. doi: 10.1101/gr.234443.118 (PMC6442396; doi:10.1101/gr.234443.118)
Supplement: Supplemental Material [file supp_gr.234443.118_Supplemental_longranger-2.2.zip › longranger-2.2.tar.bz2!/lr2.2-public/lib/stan/cmdstan/stan/doc/api/html/bare__type__grammar__def_8hpp_source.html]

Stan: src/stan/gm/grammars/bare\_type\_grammar\_def.hpp Source File


|  |  |
| --- | --- |
|  | Stan  2.5.0  probability, sampling & optimization |


- Main Page
- Related Pages
- Namespaces
- Classes
- Files

- File List
- File Members

All Classes Namespaces Files Functions Variables Typedefs Enumerations Enumerator Friends Macros Pages

- src
- stan
- gm
- grammars

bare\_type\_grammar\_def.hpp

Go to the documentation of this file.

1 #ifndef STAN\_\_GM\_\_PARSER\_\_BARE\_TYPE\_GRAMMAR\_DEF\_HPP\_\_

2 #define STAN\_\_GM\_\_PARSER\_\_BARE\_TYPE\_GRAMMAR\_DEF\_HPP\_\_

3

4 #include <boost/spirit/include/qi.hpp>

5 #include <boost/lexical\_cast.hpp>

6 #include <boost/fusion/include/adapt\_struct.hpp>

7 #include <boost/fusion/include/std\_pair.hpp>

8 #include <boost/config/warning\_disable.hpp>

9 #include <boost/spirit/include/qi\_numeric.hpp>

10 #include <boost/spirit/include/phoenix\_core.hpp>

11 #include <boost/spirit/include/phoenix\_function.hpp>

12 #include <boost/spirit/include/phoenix\_fusion.hpp>

13 #include <boost/spirit/include/phoenix\_object.hpp>

14 #include <boost/spirit/include/phoenix\_operator.hpp>

15 #include <boost/spirit/include/phoenix\_stl.hpp>

16 #include <boost/spirit/include/support\_multi\_pass.hpp>

17 #include <boost/tuple/tuple.hpp>

18 #include <boost/variant/apply\_visitor.hpp>

19 #include <boost/variant/recursive\_variant.hpp>

20

21 #include <boost/spirit/include/version.hpp>

22 #include <boost/spirit/include/support\_line\_pos\_iterator.hpp>

23

24 #include <stan/gm/ast.hpp>

25 #include <stan/gm/grammars/bare\_type\_grammar.hpp>

26 #include <stan/gm/grammars/whitespace\_grammar.hpp>

27

28 BOOST\_FUSION\_ADAPT\_STRUCT(stan::gm::expr\_type,

29  (stan::gm::base\_expr\_type, base\_type\_)

30  (size\_t, num\_dims\_) );

31

32 namespace stan {

33

34  namespace gm {

35

36  template <typename Iterator>

37  bare\_type\_grammar<Iterator>::bare\_type\_grammar(variable\_map& var\_map,

38  std::stringstream& error\_msgs)

39  : bare\_type\_grammar::base\_type(bare\_type\_r),

40  var\_map\_(var\_map),

41  error\_msgs\_(error\_msgs)

42  {

43  using boost::spirit::qi::\_1;

44  using boost::spirit::qi::char\_;

45  using boost::spirit::qi::eps;

46  using boost::spirit::qi::lexeme;

47  using boost::spirit::qi::lit;

48  using boost::spirit::qi::no\_skip;

49  using boost::spirit::qi::\_pass;

50  using boost::spirit::qi::\_val;

51

52  using boost::spirit::qi::labels::\_a;

53  using boost::spirit::qi::labels::\_r1;

54  using boost::spirit::qi::labels::\_r2;

55

56  using boost::spirit::qi::on\_error;

57  using boost::spirit::qi::fail;

58  using boost::spirit::qi::rethrow;

59  using namespace boost::spirit::qi::labels;

60

61  bare\_type\_r.name("bare type");

62  bare\_type\_r

63  %=

64  type\_identifier\_r

65  >> array\_dims\_r

66  ;

67

68  type\_identifier\_r

69  %=

70  lit("void")[\_val = VOID\_T]

71  | lit("int")[\_val = INT\_T]

72  | lit("real")[\_val = DOUBLE\_T]

73  | lit("vector")[\_val = VECTOR\_T]

74  | lit("row\_vector")[\_val = ROW\_VECTOR\_T]

75  | lit("matrix")[\_val = MATRIX\_T]

76  ;

77

78  array\_dims\_r

79  %=

80  eps[\_val = 0]

81  >> - ( lit('[')[\_val = 1]

82  >> \*(lit(',')[\_val += 1])

83  >> lit(']') )

84  ;

85

86  }

87  }

88 }

89 #endif

stan::gm::bare\_type\_grammar::bare\_type\_grammar

bare\_type\_grammar(variable\_map &var\_map, std::stringstream &error\_msgs)

**Definition:** bare\_type\_grammar\_def.hpp:37

stan::gm::bare\_type\_grammar::array\_dims\_r

boost::spirit::qi::rule< Iterator, size\_t(), whitespace\_grammar< Iterator > > array\_dims\_r

**Definition:** bare\_type\_grammar.hpp:45

stan::gm::bare\_type\_grammar::bare\_type\_r

boost::spirit::qi::rule< Iterator, expr\_type(), whitespace\_grammar< Iterator > > bare\_type\_r

**Definition:** bare\_type\_grammar.hpp:35

bare\_type\_grammar.hpp

stan::gm::DOUBLE\_T

const int DOUBLE\_T

**Definition:** ast.hpp:66

stan::gm::ROW\_VECTOR\_T

const int ROW\_VECTOR\_T

**Definition:** ast.hpp:68

stan::gm::bare\_type\_grammar::type\_identifier\_r

boost::spirit::qi::rule< Iterator, base\_expr\_type(), whitespace\_grammar< Iterator > > type\_identifier\_r

**Definition:** bare\_type\_grammar.hpp:40

stan::gm::variable\_map

**Definition:** ast.hpp:439

stan::gm::VECTOR\_T

const int VECTOR\_T

**Definition:** ast.hpp:67

stan::gm::MATRIX\_T

const int MATRIX\_T

**Definition:** ast.hpp:69

stan::gm::expr\_type

**Definition:** ast.hpp:74

BOOST\_FUSION\_ADAPT\_STRUCT

BOOST\_FUSION\_ADAPT\_STRUCT(stan::gm::expr\_type,(stan::gm::base\_expr\_type, base\_type\_)(size\_t, num\_dims\_))

ast.hpp

stan::gm::base\_expr\_type

int base\_expr\_type

**Definition:** ast.hpp:60

whitespace\_grammar.hpp

stan::gm::bare\_type\_grammar

**Definition:** bare\_type\_grammar.hpp:19

stan::gm::INT\_T

const int INT\_T

**Definition:** ast.hpp:65

stan::gm::VOID\_T

const int VOID\_T

**Definition:** ast.hpp:64


---

[ Stan Home Page ]

*© 2011–2014,
Stan Development Team.*
